# Damage Assessment of Glass-Fibre-Reinforced Plastic Structures under Quasi-Static Indentation with Acoustic Emission

**DOI:** 10.3390/ma16145036

**Published:** 2023-07-17

**Authors:** Norman Osa-uwagboe, Amadi Gabriel Udu, Vadim V. Silberschmidt, Konstantinos P. Baxevanakis, Emrah Demirci

**Affiliations:** 1 Wolfson School of Mechanical, Electrical, and Manufacturing Engineering, Loughborough University, Loughborough LE11 3TU, UK; n.osa-uwagboe@lboro.ac.uk (N.O.-u.); k.baxevanakis@lboro.ac.uk (K.P.B.); e.demirci@lboro.ac.uk (E.D.); 2 Air Force Research and Development Centre, Nigerian Air Force Base, Kaduna 800282, Nigeria; agu1@leicester.ac.uk; 3 School of Engineering, University of Leicester, Leicester LE1 7RH, UK

**Keywords:** quasi-static indentation, fibre-reinforced plastics, acoustic emission, damage, X-ray micro computed tomography, scanning electron microscopy

## Abstract

The use of fibre-reinforced plastics (FRPs) in various industrial applications continues to increase thanks to their good strength-to-weight ratio and impact resistance, as well as the high strength that provides engineers with advanced options for the design of modern structures subjected to a variety of out-of-plane impacts. An assessment of the damage morphology under such conditions using non-destructive techniques could provide useful data for material design and optimisation. This study investigated the damage mechanism and energy-absorption characteristics of E-glass laminates and sandwich structures with GFRP face sheets with PVC cores under quasi-static indentation with conical, square, and hemispherical indenters. An acoustic emission (AE) technique, coupled with a k-means++ pattern-recognition algorithm, was employed to identify the dominant microscopic and macroscopic damage mechanisms. Additionally, a post-mortem damage assessment was performed with X-ray micro computed tomography and scanning electron microscopy to validate the identified clusters. It was found that the specific energy absorption after impact with the square and hemispherical indenters of the GFRP sandwich and the plain laminate differed significantly, by 19.29% and 43.33%, respectively, while a minimal difference of 3.5% was recorded for the conical indenter. Additionally, the results obtained with the clustering technique applied to the acoustic emission signals detected the main damaged modes, such as matrix cracking, fibre/matrix debonding, delamination, the debonding of face sheets/core, and core failure. The results therefore could provide a methodology for the optimisation and prediction of damage for the health monitoring of composites.

## 1. Introduction

Composite materials have been used increasingly in various industrial applications in the past 50 years thanks to their inherent advantage of a greater stiffness-to-weight ratio than that of traditional metals. A popular variant 36 of composite materials is fibre-reinforced plastic sandwich structures (FRPSS), made from thermosetting resins due to their ease of manufacture, buoyancy, and acceptable impact resistance performance [1,2]. These materials are commonly made of glass fibre-reinforced plastics (GFRP) or carbon fibre-reinforced plastics (CFRP), and foam cores, whose performances are also affected by moisture ingress. However, the overall performance of FRPSSs makes them suitable for the construction of both above-water and underwater marine vessels [3,4,5]. These applications can expose the structures to low-velocity impact (LVI), which can produce barely visible impact damage (BVID) and lead to catastrophic failure. Several studies have adopted different measures for BVID in composite materials in order to comprehend the damage modes pertinent to these types of loading conditions [6]. Owing to the similarities in damage scenarios, it was demonstrated that a quasi-static indentation (QSI) method could give indications of the damage modes in LVI for composites [7,8]. Zniker et al. used this method to compare the energy-absorption capability of GFRP laminates and PVC-foam sandwich structures under repeated impacts and the reduced levels of energy using a modified Charpy test and QSI experiments [9]. The obtained results revealed that while the indentation energy for the laminates with varying thicknesses was identical, the presence of the foam core significantly improved the damping properties of the sandwich structures. Furthermore, the damaged area in the sandwich structures was larger than that of the laminates and predominantly characterised by delamination. Other studies showed that failure modes of sandwich structures due to QSI are similar to those caused by LVI and include core buckling, core crushing, delamination in the face sheets, and debonding between the core and face sheets, as well as matrix cracking and fibre breakage in the face sheets [10,11,12,13].

Considering that damage evolution is a critical component of structural health monitoring in composites, several non-destructive techniques (NDT), such as acoustic emission (AE), were adopted because they can monitor internal damage and provide useful information on the damage evolution process in real time [14,15,16]. Dikshit et al. successfully utilised AE techniques to characterise the energy-absorption properties of FRPSSs with varying core designs. The results showed that various damage modes (fibre breakage, fibre/matrix debonding) were obtained, and a correlation between the AE features and the corresponding absorption properties of the various cores was established [17]. Hajikhani et al. applied the AE technique to assess the strain energy release rate in mode I delamination in FRPSS with woven GFRP face sheets and a foam core. It was shown that AE signals coupled with results from mechanical tests accurately described the fracture toughness, as well as the in-plane fibre orientation effect on the fracture behaviour [18]. Further, Ben Ammar et al. used AE coupled with clustering techniques to identify and characterise the local damage in FRPSS with PVC closed-cell foam cores with different densities under a quasi-static loading regime; the obtained results demonstrated a good agreement with the experimental data [19]. Other studies also adopted the approach of utilising AE signals and clustering or pattern recognition techniques for damage analysis in composite materials [20,21,22,23]. Hence, the AE technique and the cluster analysis are a reliable methodology for the monitoring and evaluation of the damage-evolution process and the recognition of the distinctive damage modes. However, it is worth noting that a better understanding of the internal structural characteristics can be obtained post mortem, using other NDT techniques. A common approach is the use of X-ray micro computed tomography (μ-CT), as demonstrated in [15,24]. Therefore, a combination of the above processes can provide a clearer picture of the damage sequence in FRPSSs and an understanding of the phenomenological progression of damage. For instance, [25] investigated the indentation response of sandwich structures to various indenter geometries and was able to capture the constituent failure modes accurately.

To gain a broader perspective for optimising the component design and ongoing structural health monitoring, it is important to investigate the damage mechanisms and energy-absorption capabilities of composite materials, especially laminates and sandwich structures. Thus, in this paper, a machine-learning (ML) model was applied to analyse the AE features, supporting a comprehensive understanding of the damage characteristics of GFRP laminates and sandwich structures. The proposed methodology could provide valuable insights into the optimisation of composites and prediction of damage with their health monitoring in real-life applications. To this end, this study compared the damage sequence of GFRP laminates (GL) and GFRP sandwich panels (GS) using AE to identify their damage modes.

## 2. Experimental Procedure

**Figure 1:**
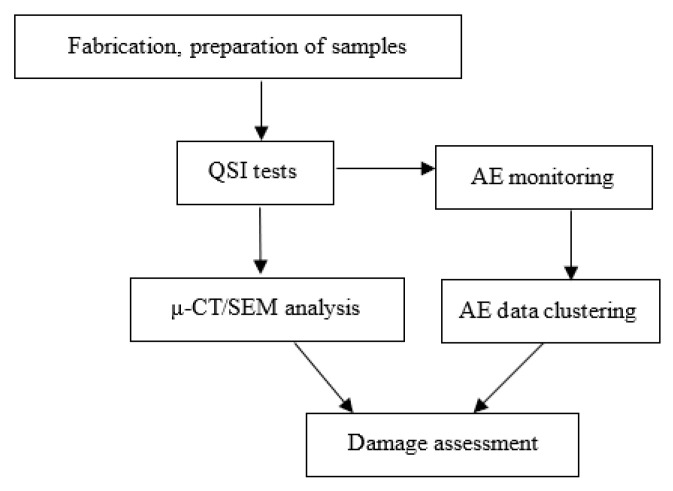
Schematic of experimental setup.

A schematic of the experimental setup used in this study is presented in Figure 1. As stated, two different sample types, GL and GS, were tested and subjected to QSI with three different indenter shapes, monitored with AE to capture visible and invisible damage in the specimens. The average data were obtained and damage assessment was conducted with a clustering algorithm, as well as μ-CT/SEM investigations.

### 2.1. Materials and Manufacturing Methods

**Figure 2:**
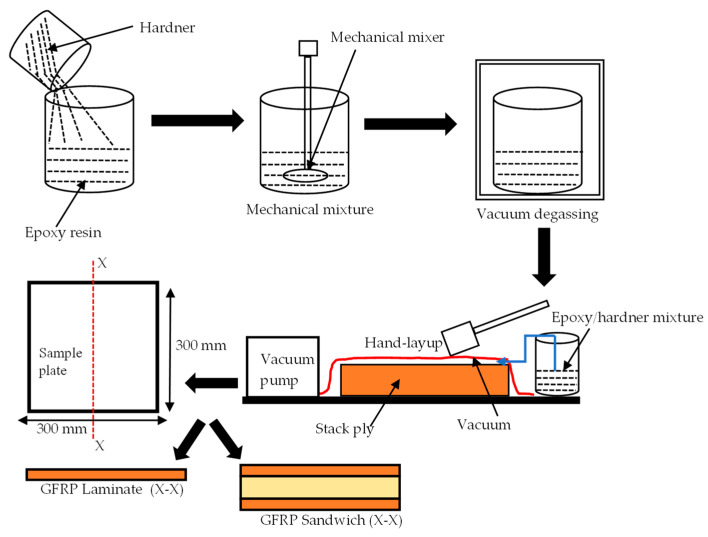
Schematic of sample fabrication process.

Aerospace-grade E-glass plain weave fabric with 160 g/m^2^ from Samson Composites Ltd. (Shenzhen, China) was used as reinforcement and face sheets for the FRPSS, while epoxy resin with C-1 catalyst hardener from EPOCHEM Ltd. (Lagos, Nigeria) with a volumetric ratio of 2:1 was employed as the matrix system to fabricate samples at room temperature (27 °C in Nigeria) with a curing time of 18 h. For the core, EASYCell 75 closed-cell PVC foam core forms from EASY composites (Stoke-on-Trent, UK) were used [26]. Details of the constituents’ parameters are given in Table 1. A hand lay-up and a vacuum-bagging technique, as described in Figure 2, were deployed to produce plates of 300 mm × 300 mm with a configuration of 8 layers of E-glass fabric for the GL samples and 4 layers/PVC foam/4 layers for the GS specimen. In order to reduce the presence of air bubbles in the mixture of the hardener and resin, it was necessary to degas the mixture for about 2 min before applying it to the sample and subsequent vacuum bagging. This mode of fabrication was selected due to its low cost, simplicity, and wide range of applicability.

### 2.2. Quasi-Static Indentation Tests

**Figure 3:**
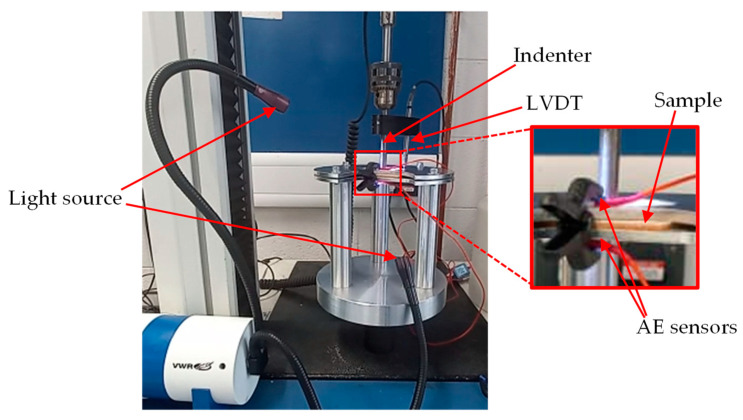
QSI experimental setup.

**Figure 4:**
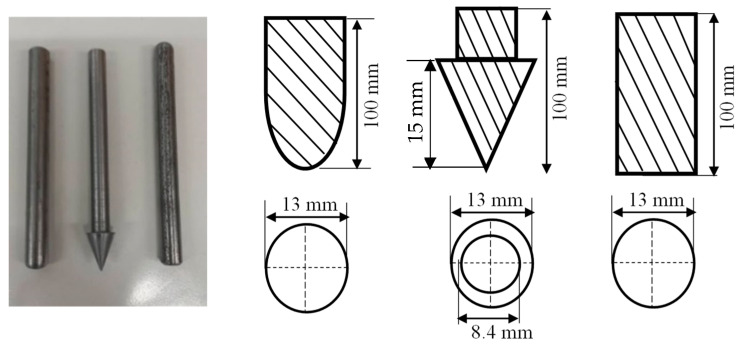
Indenter types and dimensions.

QSI was performed on the samples with different indenter shapes to study the damage tolerance of the materials, to provide useful data on the sequence of damage, with a minimum of 5 samples per configuration. The experiment was carried out in line with the ASTM D6264/D6264M-17 standard test method with a displacement control of 1 mm/min, while the vertical displacement was measured using a linear variable differential transformer (LVDT) [30]. The test was performed using an Instron 3369 Universal machine 50 kN cell (Instron Corporation, Norwood, MA, USA) with a fixture made from steel plates. Three different indenter types made from stainless steel were used for this experiment: hemispherical, conical, and flat indenters with a diameter/length of 13 mm. During the test, the indenter was aligned to the centre of the specimen with an offset at no more than 0.01 mm, and then indented until complete perforation. The experimental jig setup is depicted in Figure 3, while the indenter geometry is shown in Figure 4.

Under out-of-plane loading conditions, energy absorption is an important parameter to understand the damage evolution in composites. Therefore, a quantitative comparison of the energy-absorption properties of the samples under various indenter configurations was performed based on the following expression:

(1) $$E_{a}=\int_{x_{0}}^{x_{1}}F\left(x\right)dx$$

where $E_{a}$ is the total energy absorbed, obtained by integration of the area under the force-displacement curve. Thereafter, the specific energy absorption (SEA), which relates to the energy absorbed per area density (A), could be calculated as follows:

(2) $$SEA=\frac{E_{a}}{A}$$

All samples were codified depending on the indenter geometries and the loading regimes for the ease of identification. For example, the specimens denoted by GLH and GSC represent glass laminate indented by hemispherical indenter, and glass sandwich indented by a conical punch, respectively. The complete specimen nomenclature is given in Table 2.

### 2.3. Acoustic Emission

#### 2.3.1. Experimental Setup

**Figure 5:**
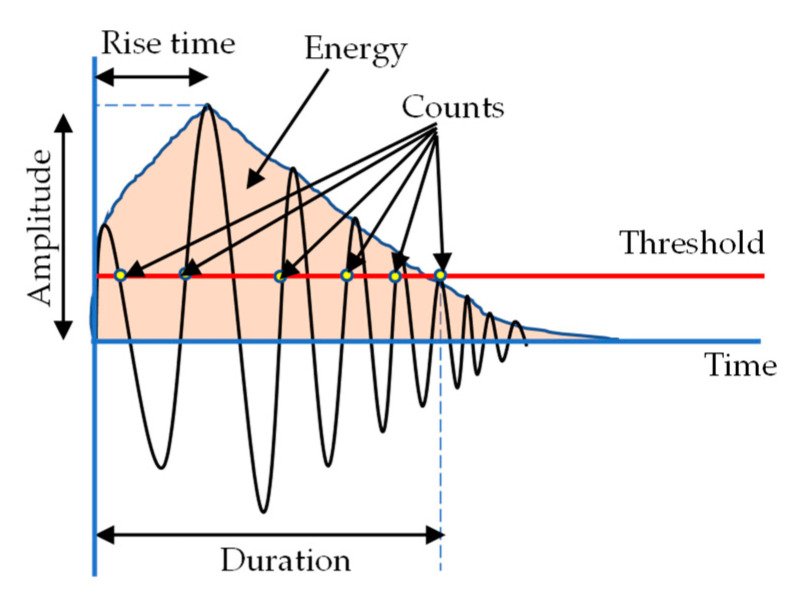
AE signal parameters.

**Figure 6:**
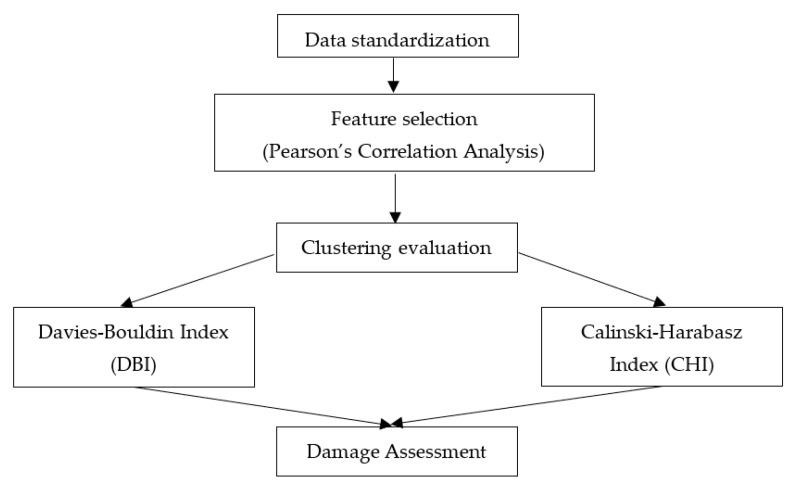
ML framework for damage assessment.

In total, 18 signal parameters, including the time and frequency domain features, were retrieved from the AE. These include Time (s), Class ID, Channel, Parametric, Risetime, Counts to Peak, Counts, Energy (J), Duration (s), Amplitude (dBae), ASL, Threshold, Average Frequency (Hz), RMS, Signal Strength, Absolute Energy (J), Frequency Centroid, and Peak Frequency (Hz). A typical description of an AE waveform and relevant parameters are shown in Figure 5. These features were utilised by the ML algorithm to assess the damage. A three-step approach was adopted for this task, namely, data standardisation, feature selection, and clustering analysis, as shown in Figure 6. A Micro-SHM system with a frequency band of 1 kHz–1 MHz (Physical Acoustics Corporation, Township, NJ, USA) with 4 AE channels and 2 parametric channels was used. However, only two channels connected to the Nano-30 AE sensors (125 kHz–750 MHz), mounted on the top and bottom face sheets (Figure 3) were used in this study, and the peak amplitude vs. time was obtained.

#### 2.3.2. Data Standardisation, Feature Selection, and Cluster Analysis

In order to exclude the dominance problems and prevent calculation complexities for the features retrieved from the AE signal parameters, data standardisation was undertaken prior to other ML tasks. All features (i.e., $x={\left(x_{1},\ldots ,x\_n\right)}^{T\;}{\epsilon R}^{\left(N\;x\;M\right)}$) were centred and scaled independently, with the standard score sample calculated using $z=\left(x-\mu \right)/\sigma ,$ where μ and σ are the mean and the standard deviation of the training data while x represents the original feature value of the matrix X. This ensured that the means were within a (0–1) range. Features can be categorised as relevant, irrelevant, and redundant. Accordingly, the inclusion/exclusion of features influences the performance of the ML algorithm. Generally, it is a challenge to ascertain which features would optimise ML algorithms’ performances for damage assessment. Hence, the feature selection techniques were employed with a view to choosing a subset from an original variable that best represents the underlying pattern, concept, or constructs investigated in the analysis. By reducing/excluding redundant and irrelevant features, ML algorithms are less likely to be misled into making a decision based on noise. Also, removing irrelevant features circumvents overfitting problems, reduces the computational cost, and enhances model accuracy [31,32,33]. In this study, feature selection was utilised to determine the quality of clustering through expert judgment and Pearson’s correlation coefficient (PCC). Correlation between data points *x* and *y* is the measure of the linear relationship between 2 features, A and B. PCC takes values ranges of ±1, where a value of zero represents the lack of linear correlation while −1 and +1 stand for perfect negative and positive correlation, respectively. PCC is adept in feature selection since it is based on the method of covariance and was employed in the literature for damage analysis [34,35]. PCC is defined as:

(3) $$r=\frac{\sum_{i=1}^{n}\left(x_{i}-\bar{x})(y_{i}-\bar{y}\right)}{\sqrt{\sum_{i=1}^{n}{\left(x_{i}-\bar{x}\right)}^{2}\sum_{i=1}^{n}{\left(y_{i}-\bar{y}\right)}^{2}}}$$

where *n* is the total number of samples, while $\bar{x}\;and$ $\bar{y}$ are the average values of input data *x_i_* and *y_i_*, respectively. Furthermore, this study adopted peak frequency and the amplitude for subsequent damage assessment. Previous studies [35,36] showed that some AE features provided more reliable data for assessing damage, with the peak frequency being the most appropriate since it is not likely to be affected by attenuation, while the amplitude was computed to have the lowest PCC score among other time-domain features. Therefore, out of the 18 features, only 7 were analysed using AE.

Two cluster validity indices, the Calinski–Harabasz index (CHI) and the Davies–Bouldin index (DBI), were adopted to estimate the number of clusters for damage assessment. These indices were combined in previous studies in estimating the optimal number of clusters for damage assessment in AE [14,35,37]. CHI gives a ratio of the degree of separation between clusters to the degree of inter-cluster dispersion, with higher CHI scores suggesting better results of well-separated and tightly packed clusters. Mathematically, CHI is defined as:

(4) $$CHI=\frac{B}{W}\cdot \frac{n_{X}-k}{k-1}$$

where *B* and *W* are the between and inter-cluster variances, respectively, while *_X_* is the data of size $n_{X}$ clustered into *k*. Based on DBI, well-separated clusters with low intra-cluster variance have higher scores than tightly packed clusters with high intra-cluster variance. DBI is defined as:

(5) $$DBI=\frac{1}{k}\sum_{m=1}^{k}\begin{array}{c} max\\ n\neq m \end{array}\left(\frac{P_{m}+P_{n}}{D_{mn}}\right)$$

where k is the number of clusters, while $D_{mn}$ is the distance between the centroids of clusters m and n; $P_{\left(m,n\right)}$ is the average distance between all points in cluster m and the centroid of cluster n. Low DBI implies a dense and well-separated cluster. Furthermore, this study employed the k-means++, an unsupervised learning algorithm that minimised the distance between vectors in the cluster. The algorithm randomly selects a single point from the AE dataset as the first cluster centre and iteratively selects the remaining cluster centres by choosing new centres that are far away from the previously chosen ones. Once all defined numbers of centres have been selected, the algorithm assigns each data point to its nearest centre and recalculates the centres based on the new assignments. This process is repeated until there is no significant change in assigning data points to clusters. The optimum number of clusters is indicated by the cluster validity combined with the least separation. It is worth noting that AE signals comprise volumetric information for deformation and damage in the specimens. A one-to-one correlation of acoustic signals with specific damage events and location was not attempted in this study.

### 2.4. Damage Characterisation

#### 2.4.1. X-ray μ-CT

**Figure 7:**
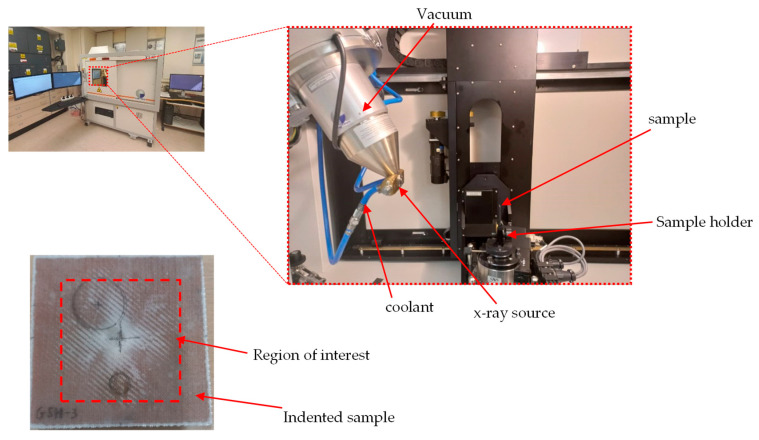
X-ray μ-CT experimental setup.

The samples were scanned using a high-resolution X-ray μ-CT system NIKON XTH X-Tex 160Xi, (NIKON Metrology Europe, Leuven, Belgium) with an effective pixel size of 27.79 μm. The beam energy, beam current, and power settings were 65 kV, 65 μA, and 4.2 W, respectively, with an exposure time of 500 ms. A total of 3016 tiff images were created per sample scan, with the region of interest limited to the areas surrounding the damaged portion of the samples, as can be seen in the experimental setup in Figure 7. The acquired microscopy data were first processed with VG Studio Max 3.1 software (Volume Graphics, Charlotte, NC, USA) and then post-processed using commercial software (Dragonfly ORS, Adelaide, South Australia, Australia). The optimum centre of rotation was determined in the initial post-processing phase, with all generated slices combined to develop the volumetric image in the reconstruction phase. Gaussian filters were used to reduce the characterisation noise generated by the volumetric imaging, which led to a minimal data loss from the sample materials’ homogeneity. The generated tiff files were incorporated into the Dragonfly software with voxel analysis to determine damage parameters.

#### 2.4.2. SEM Analysis

Before conducting SEM analysis, the samples were sputter-coated with Au/Pd with a coating thickness of about 6 nm, then images were captured with a JSM-7800F SEM machine (UK) at an acceleration voltage of 10 kV and probe current of 200 pA. Image post-processing was carried out using Aztec software (Oxford Instruments, Oxford, UK), and the damage mechanism was accurately identified.

## 3. Results and Discussion

### 3.1. Load–Displacement Results

**Figure 8:**
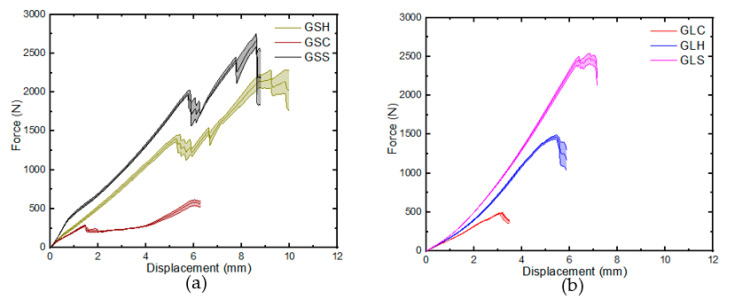
Load–displacement curves: (**a**) GS, (**b**) GL.

The average displacement curve for the GS and GL samples for the different indenter geometries are shown in Figure 8. In general, the samples exhibited quasi-brittle failure in three identifiable stages, with two noticeable peaks for the GS samples and a single peak for the GL samples in the load–displacement graphs. These peaks corresponded to the failure of the top and bottom face sheets in the GS samples and laminate failure in the GL specimen. A similar trend of peak forces in the load displacement curves representing the damage characteristics of FRP were reported in [23,24]. For the GS samples described in Figure 8a, it was observed that the load increased in a similar pattern up to the fracture of the bottom face sheets at 2.67 kN, 2.17 kN, and 0.58 kN for GSS, GSH, and GSC, respectively. This variation could be attributed to the difference in the surface area in contact with the sample, as a reduction in the load was required for the penetration of the top face sheet as the indenter angle in contact with the specimen reduced. Consequently, a smaller force was necessary for penetration as the indenter progressed through the sample thickness. The GSC specimen exhibited the top face sheet penetration at a displacement of 1.5 mm, representing 28.3% and 26.3% of the displacement required for the corresponding face sheet penetration in GSH and GSS. Additionally, the penetration load was significantly lower at 79.9% and 85.9% of the force required for GSH and GSS, respectively. After the drop in the load at the first peak, there was a rise in load due to the contribution of the bottom face sheet resistance and the friction of the core as the indenter progressed through the thickness of the sample until the fracture of the bottom face sheet.

For the GL samples (Figure 8b), a similar quasi-brittle behaviour was observed as the load rapidly increased until the fracture of the fibres at 2.48 kN, 1.49 kN, and 0.48 kN for the GLs, GLh, and GLc specimens, respectively. The limited surface area of contact between the impacted material and the conical indenter, observed for GS samples and which resulted in smaller loads, might also have a similar effect on the laminate samples. It is noteworthy that while the load–displacement curve for the GS specimens considers the contribution of the core and bottom face sheets, the corresponding curve for the laminates was similar to the portion of the GS samples that related to the top face sheets with comparable thickness.

### 3.2. Energy-Absorption Properties

**Figure 9:**
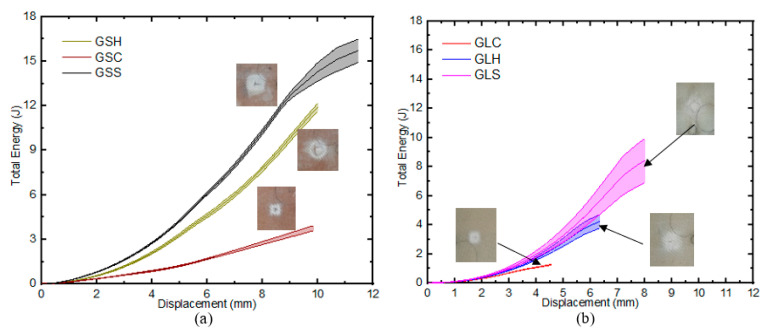
Total energy analysis: (**a**) GS, (**b**) GL.

The total energy absorbed by all samples with the conical indenter was generally lower when compared to other indenter geometries (Figure 9). The GLC absorbed 13.3% and 28.0% of the energy of GLS and GLH before fracture, respectively. Similarly, GSC absorbed 14.1% and 17.7% of the energy of GSS and GSH before fracture, respectively. It is worth noting that the drops noticed in the top face sheets in the force-displacement diagrams of the GS samples were small, and thus there was no noticeable decline in $E_{a}$ for the specimens.

**Figure 10:**
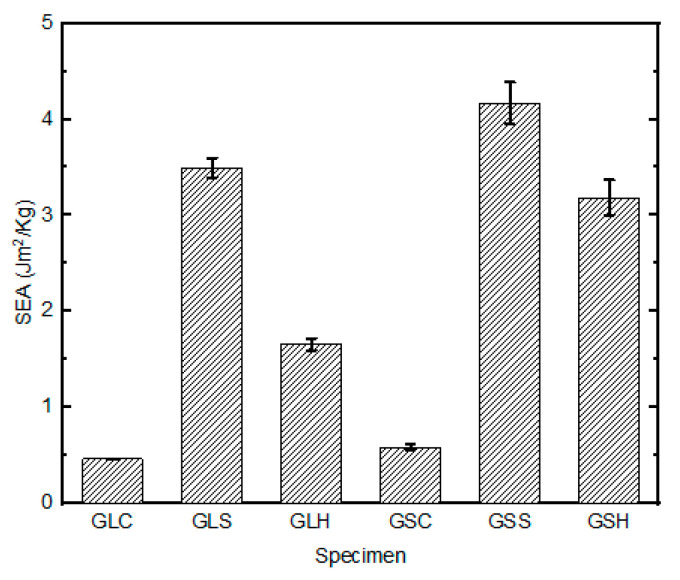
Specific energy absorption for different samples.

A comparison of the energy-absorption capabilities per unit thickness of the GS and GL samples revealed that the former (GSS and GSH) had the highest potential for energy- absorption, while the samples indented by the conical indenter (GS_c_ and GL_c_) had lower energy-absorption properties. The SEA of the GL and GS samples impacted with the square and hemispherical indenters differed significantly, by 19.2% and 43.3%, respectively (Figure 10). This is attributed to the presence of the core and the related increase in the sample thickness. However, for the samples tested under conical indentation, the energy-absorption curves showed an insignificant difference (3.5%) between the GS and GL samples. This was solely caused by the shape of the indenter, and thus limited the contributory effects of the core and through-thickness on the energy-absorption properties of the samples under such regimes.

### 3.3. Acoustic Emission

#### 3.3.1. Feature Analysis

**Figure 11:**
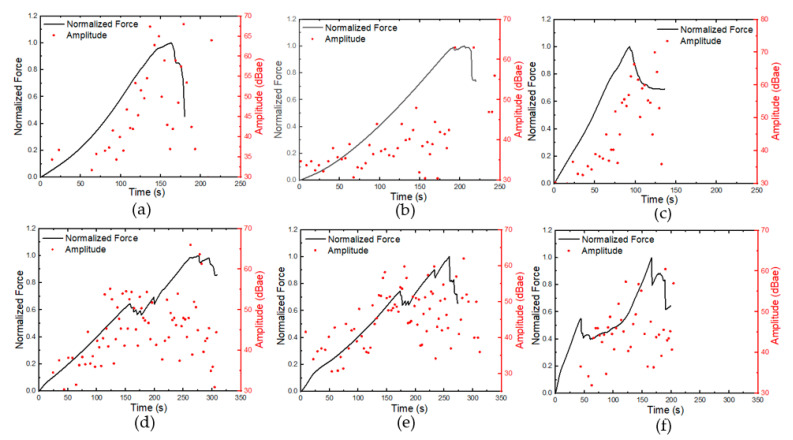
Average force-time curve with AE amplitude: (**a**) GLH, (**b**) GLS, (**c**) GLC, (**d**) GSH, (**e**) GSS, (**f**) GSC.

**Figure 12:**
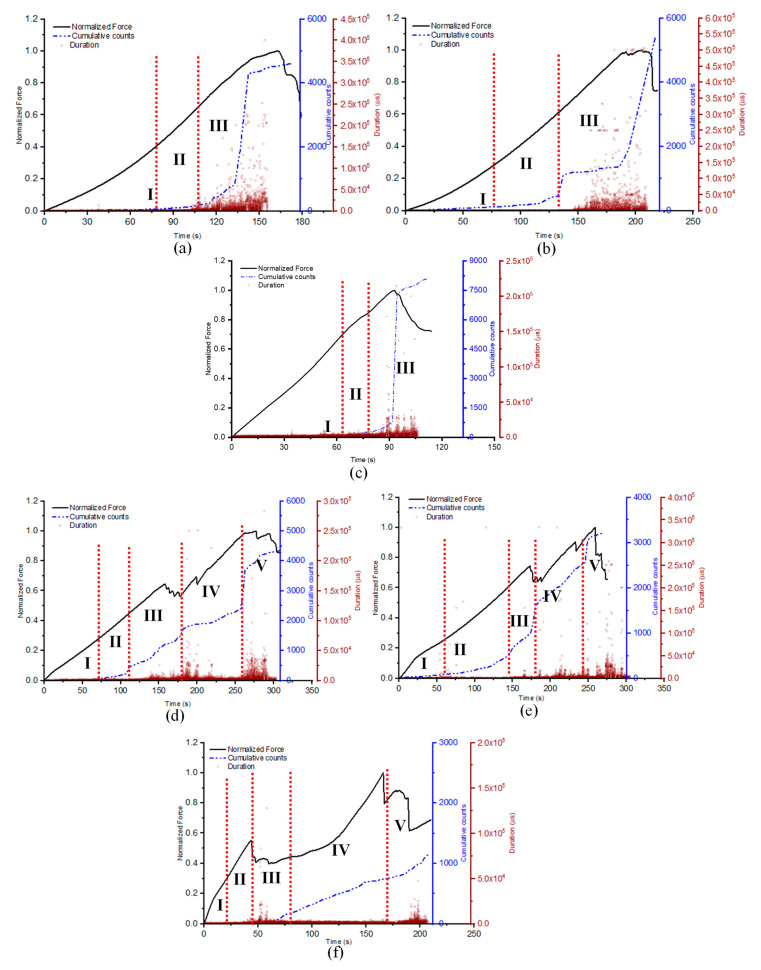
Normalised force-time curves with AE cumulative counts and duration: (**a**) GLH, (**b**) GLS, (**c**) GLC, (**d**) GSH, (**e**) GSS, (**f**) GSC.

The normalised force-time graphs of the samples under various indenter geometry with the corresponding AE signals are presented in Figure 11. The aim was to monitor the variation in AE features in order to identify failure points (drop in force) with respect to time. For the laminate samples (Figure 12a–c), crack initiation with significant plastic deformation was observed until failure, with a drop in the load curve at around 150 s, 180 s, and 100 s for GLH, GLS, and GLC, respectively. The increase in the amplitude of AE signals was attributed to crack propagation up to the critical load, beyond which the AE inputs could be regarded as noise from the experimental setup [38,39]. Similar observations were made for the GS samples (Figure 12d–f), with an increase in the amplitude of AE signals as the force progressed through two notable peaks corresponding to the top and bottom face sheet failures.

The failure of the top and bottom face sheets was characterised by higher amplitude signals (>50 dB) observed at around 150 s and 300 s for the GSH and GSS specimens, respectively. However, for the GSc specimen, higher amplitudes due to face sheet perforation occurred earlier (50 s and 150 s), which was attributed to the shape of the conical indenter, leading to lower damage resistance of the samples. As the conical indenter penetrated the sample, the contact area with the sample progressively increased from the tip to the base, resulting in the expansion of the damaged area and leading to the failure of more fibres, recorded as high amplitude signals over a longer time when compared to other GS samples. A similar indenter-induced damage recognition with AE was reported in [17,25]. It should be noted that in this study, high amplitude hits recorded prior to the onset of damage were assumed to be signals generated by the mechanical setup and not indicative of the damage mechanism in the samples.

It was proven that the AE cumulative count behaviour provided a significant role in assessing the damage mechanism and failure characteristics of composite materials as it allows a substantial classification of distinct zones of damage under quasi-static loading conditions [40]. Therefore, to further clarify the damage mechanism corroborated with AE features, it was necessary to analyse the AE counts and duration features. A change in the gradient of the cumulative counts slope indicated a transition in the load-bearing capacity of the sample, reflecting of the presence of a damage sequence, which resulted in a corresponding increase in the duration hits [38]. As can be seen from Figure 12a–c, three distinct regions were identified for the laminates, while 5 were established regions for the sandwich samples.

For the laminate specimens, region I was identified as the initiation of damage at the microscopic stage (matrix cracking), region II represents the initiation of macroscopic damage such as fibre/matrix debonding, and finally region III indicates the start of damage propagation until failure of the structure (fibre breakage/rupture) [39]. On the other hand, two additional damage processes could be identified (regions IV and V) for samples, and the earlier damage points discussed were similar to those of the face sheet of the GS samples [17]. Region IV represents the core/face sheet debonding and delamination, while region V indicates the fracture of the bottom face sheets. It could be seen that the duration of region II (in Figure 12a–c) for the laminates decreased with a reduction in the contact surface area of the indenter, leading to a faster penetration and subsequent macroscopic damage.

Conversely, region IV (in Figure 12d–f) for the sandwich specimens experienced a much gentler slope of the AE cumulative counts and steady duration hits with an amplitude below 50 dB, indicative of the limited damage resistance offered by the core to the conical indenter up to bottom face sheet perforation. This could be attributed to the low stiffness/thickness ratio of the foam core, thereby resulting in the bottom face sheet primarily responsible for damage resistance of the structure.

#### 3.3.2. Pattern-Recognition Analysis

**Figure 13:**
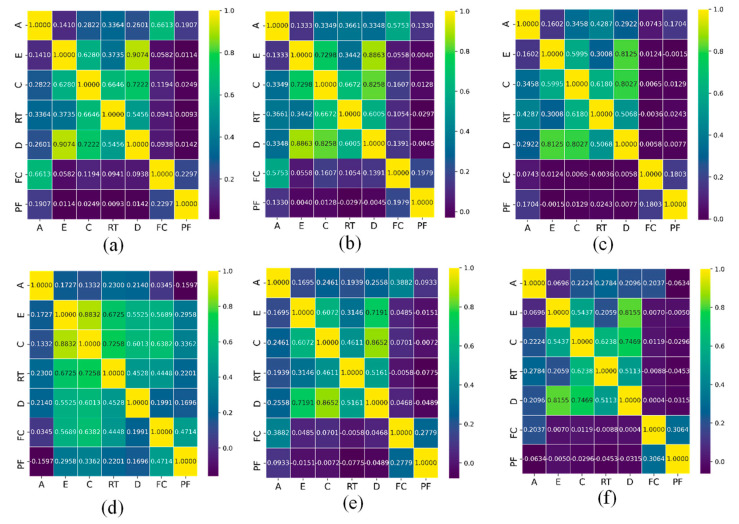
PCC between AE signals for different specimens: (**a**) GLH, (**b**) GLC, (**c**) GLS, (**d**) GSH, (**e**) GSC, (**f**) GSS (PF—peak frequency, A—amplitude, D—duration, C—counts, RT—rise time, E—energy, and FC—frequency centroid).

**Figure 14:**
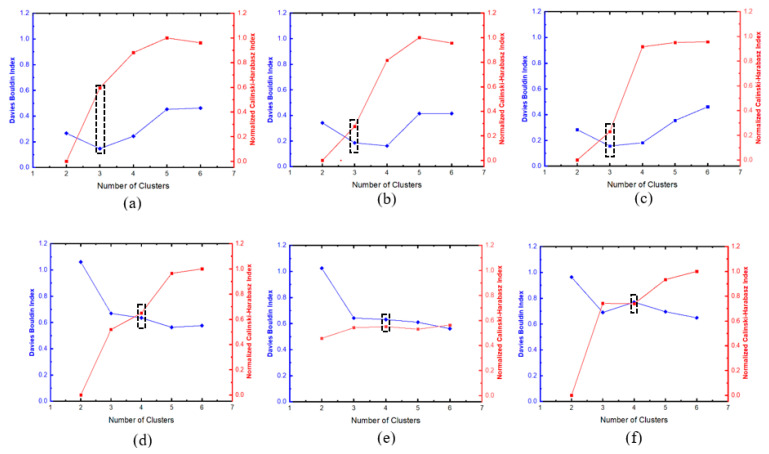
Pearson’s correlation; (**a**) GLH, (**b**) GLC, (**c**) GLS, (**d**) GSH, (**e**) GSC, (**f**) GSS.

The PCC for the selected AE features (defined in Section 2.3.2) for 6 specimens is shown in Figure 13. Among all features, the peak frequency, which is expected to be affected by attenuation, was selected for cluster analysis, together with the amplitude feature with the lowest time-domain PCC. The results for the PCC for the GS and GL samples are provided in Figure 13. As shown in Figure 14, the k-means++ clustering analysis separates the AE data into 3 and 4 clusters, representing the damage modes for the GS and GL specimens, respectively. The cluster validity evaluation scores for different specimens are presented in Table 3.

**Figure 15:**
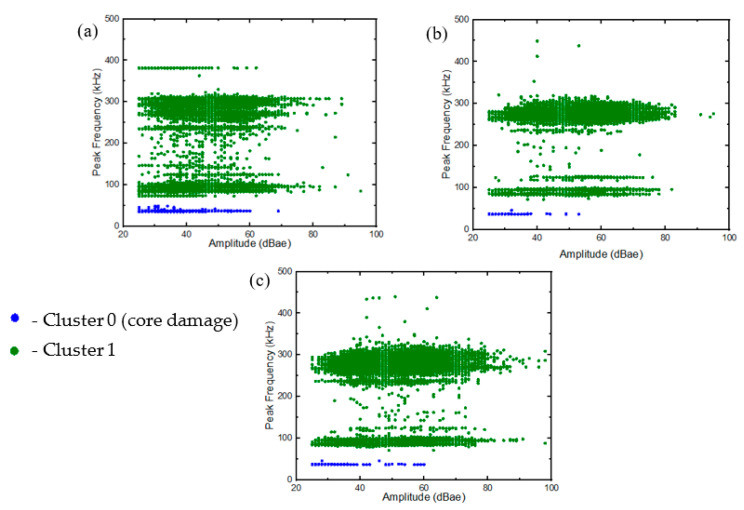
Stage 1 clustering: (**a**) GSH, (**b**) GSC, (**c**) GSS.

The two-stage clusterisation approach proposed in [39] was adopted to determine the damage morphology of the samples. The first stage for the GS specimen was conducted with AE features divided into two broad classes. As discussed earlier, the damage mechanism in the GL samples was similar to that of the face sheets of the GS specimens and, thus, the additional cluster could be attributed to the damage in the core of the GS specimen via direct elimination method. This was also corroborated by the similarities in the AE events described in Figure 11 and Figure 12. In Figure 15, cluster 0 represents the low-frequency features (<60 kHz), with a variation in amplitude (up to 60 dBae) indicative of a damage phenomenon, while cluster 1 represents features with frequencies above 75 kHz and up to 85 dBae. The frequency range for core damage can be delineated according to the previous literature [39,41].

**Figure 16:**
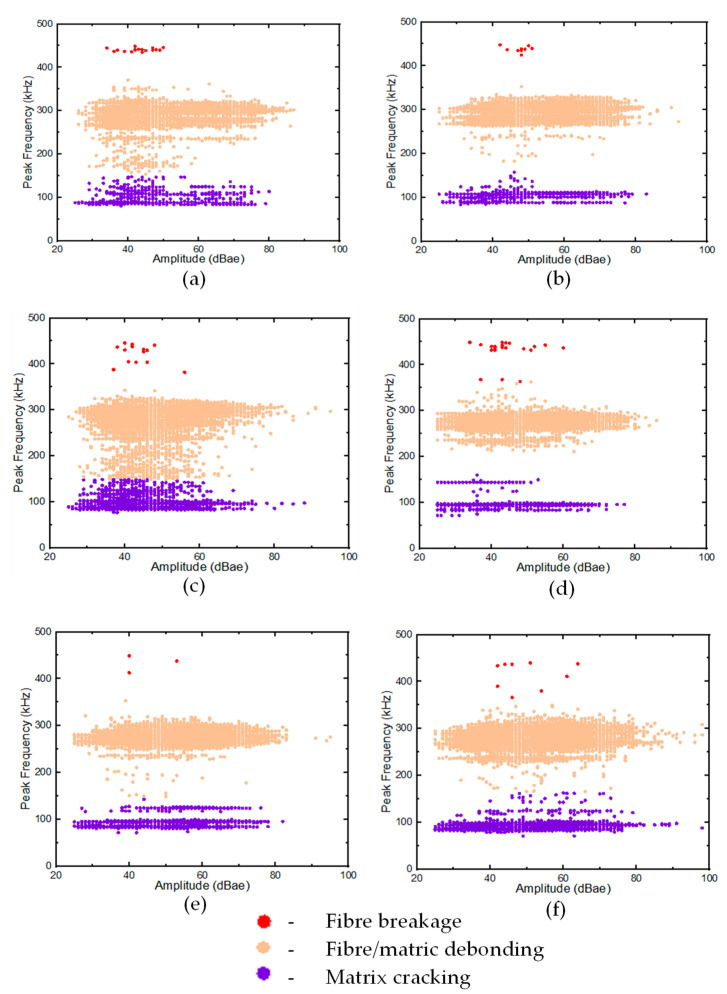
Stage 2 clustering (**a**) GLH, (**b**) GLC, (**c**) GLS, (**d**) GSH, (**e**) GSC, (**f**) GSS.

Stage 2 of the clusterisation involved the processing of all the remaining AE features in the GS and GL samples. Three distinct clusters were observed (see Figure 16), and the corresponding damage sequence was attributed to the laminates and face sheets of the GS samples. The results revealed that the frequency content attributed to matrix cracking was recorded within the range of 60–140 kHz, with most hits in the range between 90 and 110 kHz, similar to the results reported in [16,28]. This range could be observed both in GL and GS samples. Similarly, further phenomenological damage, such as fibre/matrix debonding and fibre breakage, was also obtained from the analysis. Two higher frequency damage modes identified in the tested specimens were cluster hits at 180–315 kHz and 360–453 kHz, with a concentration of hits at 222–315 kHz and 453 kHz, corresponding to fibre/matrix debonding and fibre breakage, respectively.

These results are similar to the values reported in other studies [42]. In general, the fourth damage cluster observed in the GS samples and the three clusters in the GL specimens were corroborated by the PCC analysis of the AE hits for the GS samples discussed above.

### 3.4. Damage and Failure Morphology

#### 3.4.1. X-ray μ-CT Results

**Figure 17:**
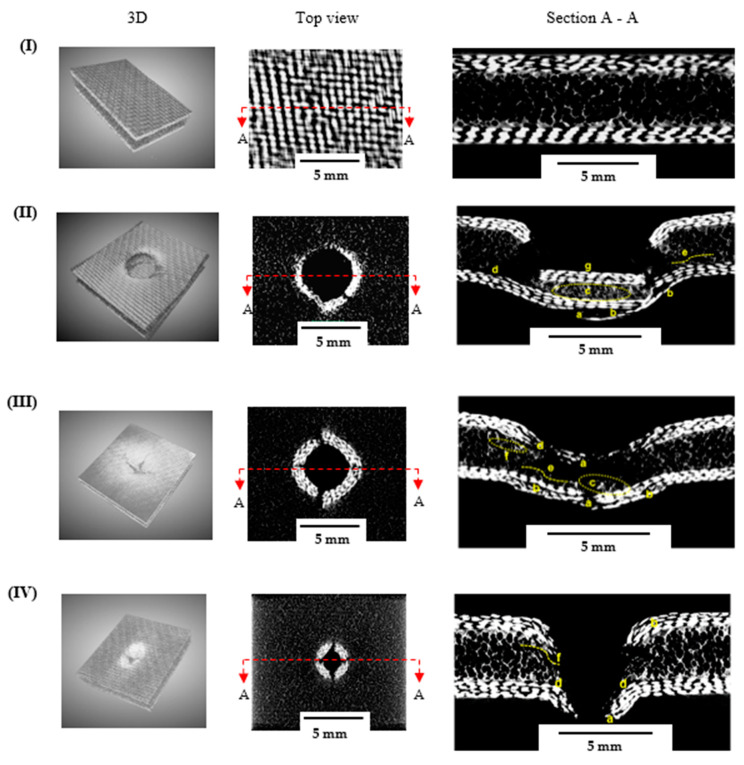
X-ray μ-CT analysis for GS samples: (**I**) undamaged (**II**) GSS, (**III**) GSH, (**IV**) GSC note; a—fibre breakage, b—delamination, c—core crushing, d—core/face sheet debonding, e—core fracture crack, f—core compression, g—compressed face sheets.

After applying the AE clusterisation technique, the identified damage morphology was validated using X-ray μ-CT and SEM images. For the μ-CT investigation, three samples per configuration were examined, and parameters such as area, perimeter, and depth were computed and presented. It has been established that understanding BVID is crucial, since out-of-plane energy is dissipated through the internal damage mechanism (such as delamination, debonding, and matrix crack) of the structures [43,44,45]. The 3D, top, and cross-sectional views of the undamaged, GSS, GSH, and GSC samples are shown in Figure 17I–IV. All images were taken at the impact region where maximum damage occurred, and various damage mechanisms were revealed for the different indenter configurations.

**Figure 18:**
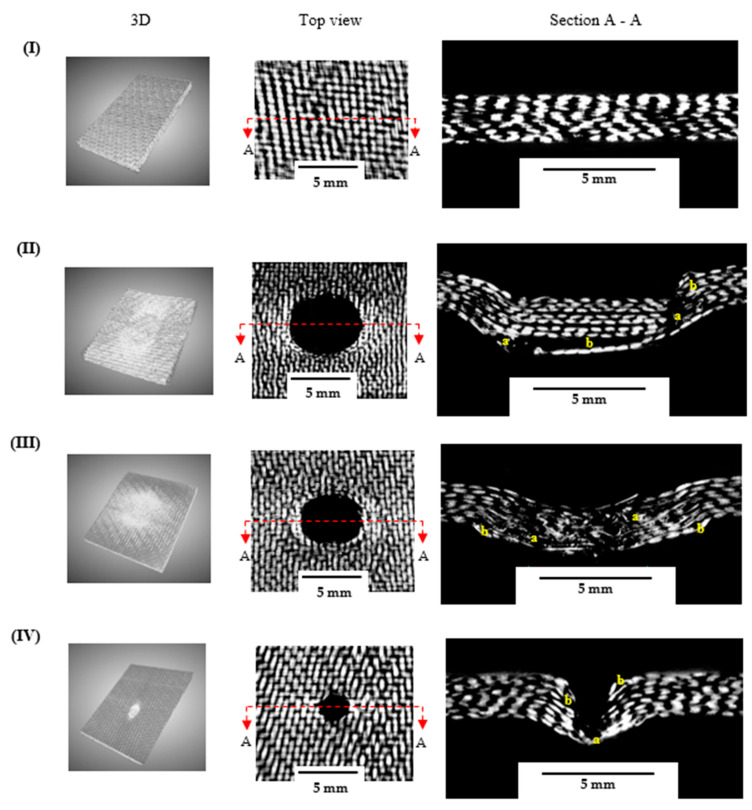
Post mortem μ-CT analysis for GL samples: (**I**) undamaged, (**II**) GLS, (**III**) GLH, (**IV**) GLC. Note: a—fibre breakage, b—delamination.

The damage evolution was more pronounced in samples indented with the hemispherical and square impactors than with the conical indenter, as evidenced by a larger damaged area. Apparently (Figure 17), the continuous loading of the sample during the QSI test caused the foam core to experience severe central crushing for the GSS and GSH samples, while all samples experienced core side shearing. For samples with a higher contact area with the indenter, the predominant damage mechanism included core crushing, delamination of the bottom face sheet, and the abrupt fibre fracture of the top face sheets. Interestingly, this fracture was identical to the indenter configuration in the GSS samples. This could also be attributed to the brittleness of the reinforcements and the high friction as the indenter moved through the specimen. Furthermore, the extent of the fibre breakage in the bottom face sheet was less severe for the GSS and GSH samples when compared to the GSC ones. This is attributed to the shape of the conical indenter; moving through the sample with a lower contact area resulted in greater penetration of the indenter as well as limited frictional resistance from the face sheet and the core. Further, a post mortem analysis of the GL samples (Figure 18) revealed damage characteristics similar to those of the GS samples impacted with the square and hemispherical indenters. Interlaminar delamination and fibre breakage were identified as the dominant damage mechanisms, while for the conical indenter damage was more localised within a smaller area, which could be attributed to the geometry of the indenter and the brittle nature of the reinforcement. It is worth noting that the damage morphology of the laminates is understandably similar to that of the top face sheet of the sandwich samples for all the indentation configurations. These were all consistent with the results obtained from the AE signal clusterisation.

Additionally, the effect of the indenter shape on the energy could be evaluated from the area, perimeter, and damage depth per thickness $\left(d/t\right)$ of damage in the samples. It was observed that the damaged area and perimeter for the square and hemispherical indenters were higher than those for the conical indenters. However, d/t was similar for almost all samples, except those indented with the square indenter, which was considerably higher, at 1.8 and 2.02 for GSS and GLS, respectively. This could be due to the shape of the indenter, which prevented easy penetration, thereby increasing the friction between the indenter and the constituents of the sample. Added to this phenomenon is the brittle nature of the fibres, which caused abrupt damage at the top face sheet and laminate (Figure 17II and Figure 18II). A summary of the damage parameters measured from the post mortem X-ray CT scan of the samples loaded with various indenter geometries is listed in Table 4.

#### 3.4.2. SEM Analysis

**Figure 19:**
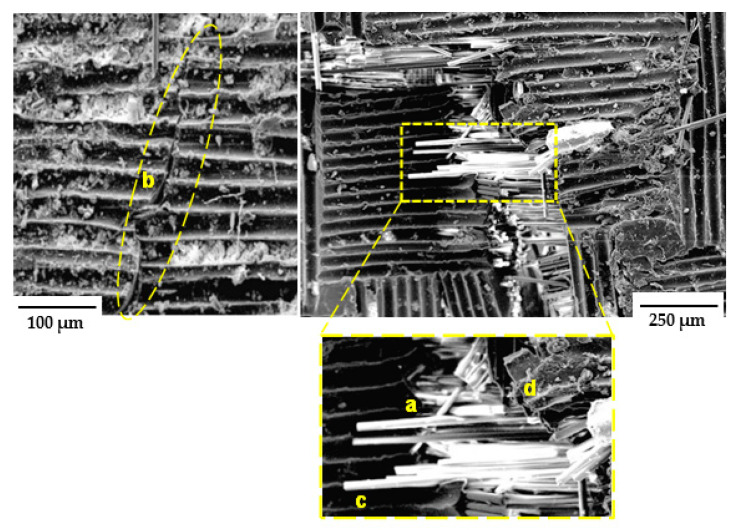
SEM analysis of GS face sheet and GL: a—fibre breakage, b—matrix crack, c—fibre pull-out, d—fibre/matrix interface debonding.

To obtain a deeper understanding of the damage morphology at the micro-level, SEM images of damaged samples taken at 250× magnification are presented in Figure 19. Higher magnification revealed that the glass fibre had a section of bare surface (Figure 19), which is indicative of the lack of adhesion between the matrix and the fibres. Furthermore, sections of the images showed a wave-like damage morphology, which indicates the presence of the matrix cracking damage mode. Thus, at the microscale, the presence of these damage modes (matrix cracking, fibre breakage, and fibre breakage with pull-out) corroborated the morphological damage phenomenon earlier observed in the μ-CT images; a similar trend was also reported by [46]. Having demonstrated the effectiveness of this methodology for exploring the damage morphology of the FRPs, it is suggested that this approach could be expanded to elucidating the energy absorption and damage sequence of FRPs subjected to extreme environmental conditions, such as seawater and Arctic temperatures.

## 4. Conclusions

The energy-absorption properties and the damage morphology of GFRP laminates and sandwich structures with the GFRP face sheet with a PVC foam core when subjected to indenters with different shapes were investigated. The experimental results obtained from the QSI tests, as well as AE monitoring, produced the following conclusions.

Under QSI conditions, the contact area with the indenter played a crucial role in the damage resistance of the GS and GL samples. For the former, the conical indenter with the surface contact exhibited lower loads at the top face sheet (28.3% and 26.3% of values for GSH and GSS samples, respectively) and complete penetration (20.1% and 14.1% of the respective values for GSH and GSS). Similar results were obtained for the GL samples, with lower loads at fracture of 2.5 kN, 1.7 kN, and 0.48 kN for the GLS, GLH, and GLC specimens, respectively. Relatedly, lower energy-absorption capabilities were presented by GSC samples (14.1% and 17.8% of the energy of GSS and GSH, respectively) and GL_c_ (13.25% and 28.03% of the energy of GL_s_ and GL_h_ before fracture, respectively).

The unsupervised pattern recognition analysis of the AE events showed that the GS had four clusters, while the GL had three clusters. These clusters were linked to the damage mechanisms identified through post mortem X-ray μ-CT and SEM studies of the samples, namely, matrix cracking, fibre/matrix debonding, core shearing/crushing, and fibre breakage. The damage-characterisation methodology proposed in this study, therefore, proved that the AE technique could underpin the understanding of failure modes in composite structures by identifying the main damage mechanisms, including those experienced by the cores of sandwich materials. The data obtained could be useful for the optimisation of the materials, while the applied methodology could be expanded to investigate damage in FRP structures subjected to varying environmental factors. Furthermore, as a future study, the effects of the varied thickness of GS and the number of layers of GL on the failure modes could be investigated with the developed AE methodology.

## Figures and Tables

**Table 1:** Mechanical properties of constituent materials [27,28,29].

Material	Young’s Modulus (GPa)	Shear Modulus (GPa)	Tensile Strength (MPa)	Poisson Ratio	Density (g/cm^3^)
	*E*	*G*_12_		$\nu_{12}$	ρ
E-glass fabric	72.39	8.27	3100–3800	0.26	2.25
PVC foam	0.075	0.028	1.89	-	0.075
Epoxy matrix	3.2–3.5	-	70–80	0.29	1.16

**Table 2:** Nomenclature of samples.

Designation	Thickness (mm)	Description
GLH	2	Laminate/hemispherical punch
GLC	2	Laminates/conical punch
GLS	2	Laminate/square punch
GSH	5	Sandwich/hemispherical punch
GSC	5	Sandwich/conical punch
GSS	5	Sandwich/square punch

**Table 3:** PCC for various samples.

Specimen	PCC	
	DBI	CHI
GLH	0.147	0.595
GLC	0.185	0.277
GLS	0.156	0.230
GSH	0.635	0.649
GSC	0.631	0.550
GSS	0.770	0.739

**Table 4:** Summary of damage parameters for GS and GL samples.

Specimen	Peak Load (N)	Max Energy (J)	Area (mm^2^)	Perimeter (mm)	d/t
GSH	2259.38 ± 71.88	12.07 ± 0.33	150.96 ± 16.94	47.09 ± 2.03	1.49 ± 0.02
GSC	589.60 ± 22.91	2.14 ± 0.10	37.61 ± 1.58	23.77 ± 0.15	1.34 ± 0.02
GSS	2774.60 ± 59.17	15.20 ± 0.71	119.59 ± 9.03	48.42 ± 1.12	1.80 ± 0.05
GLH	1488.40 ± 24.98	4.58 ± 0.19	48.04 ± 5.03	26.55 ± 1.17	1.48 ± 0.13
GLC	499.93 ± 7.58	1.28 ± 0.02	10.23 ± 1.46	13.67 ± 0.39	1.5 ± 0.09
GLS	2259.83 ± 71.88	9.70 ± 0.27	76.70 ± 9.44	32.59 ± 1.74	2.02 ± 0.08

## Data Availability

The data presented in this study are available on request from the corresponding author.
